# Frequency and Severity of Low Back Pain in Nurses Working in Intensive Care Units and Influential Factors

**DOI:** 10.12669/pjms.301.3455

**Published:** 2014

**Authors:** Ozlem Ovayolu, Nimet Ovayolu, Mehtap Genc, Nilgun Col-Araz

**Affiliations:** 1 Ozlem Ovayolu, RN, PhD, Gaziantep University, Faculty of Health Science, Gaziantep, Turkey.; 2 Nimet Ovayolu, RN, PhD, Associate Professor, Gaziantep University, Faculty of Health Science, Gaziantep, Turkey.; 3 Mehtap Genc, RN, BSN, Instructor, Bitlis Eren University, Bitlis, Turkey.; 4 Nilgun Col-Araz, Gaziantep University, Faculty of Medicine, Department of Pediatric, Gaziantep, Turkey.

**Keywords:** Low back pain, Nurse, Influencing factors, Intensive care units

## Abstract

***Objective:*** The purpose of this research was to determine the frequency and severity of low back pain and influencing factors in nurses working in intensive care units.

***Methods:*** This research was conducted as a cross-sectional study with 114 nurses working in the intensive care units in the province of Gaziantep, Turkey. Study data were collected using a questionnaire form and visual analogue scale.

***Results:*** It was found that 84.2% of the nurses experienced low back pain, and 66.7% of the nurses evaluated this pain as “a pain with moderate severity”. It was determined that nurses who had not received any education on low back pain, who remained standing for long periods of time, who performed interventions that required bending forward, who lifted and repositioned patients, and who did not use any aiding equipment during interventions, experienced more pain and had higher average pain scores. In addition, average pain scores were higher among nurses with master’s and doctorate degrees, and those working in internal medicine and pediatric intensive care units and working in shifts.

***Conclusion:*** It was observed that many of the nurses working in intensive care units experienced low back pain, and especially those working in internal medicine and pediatric intensive care units and working in shifts had higher average pain scores.

## INTRODUCTION

Nurses are among the professionals with the highest incidence rates of work-related low back problems.^1^ Because nursing interventions include physical, personal and ergonomic risk factors for low back pain.^2^^,^^3^ Due to the low back pain caused by these risk factors, every year thousands of nurses in the world work with less efficiency, receive medical reports and/or retire early. Especially the nurses working in intensive care units experience low back pain more frequently due to reasons such as providing patient care by bending forward for long durations, over-forcing/over-loading some body parts while repositioning patients, and sparing more time for patient care. In addition, over-workload in intensive care units, and frequent repetition of body movements and functions such as reaching up-forward, holding, clasping-hugging, lifting and turning prepare the ground for the emergence of this problem.^4^

Despite this fact, there is very limited number of studies on the assessment of low back pain in nurses who work in intensive care units.^4^ Studies conducted on this subject in our country are also not very common. Thus, this study aimed to determine the frequency and severity of low back pain and influencing factors in nurses working especially in intensive care units that include many of the risk factors for low back pain. In addition, it was considered important to provide suggestions at the end of this study for taking necessary precautions to reduce low back pain in nurses who work in intensive care units.

## METHODS

***Design and sample:*** A cross-sectional and descriptive design was used in this study that was conducted with a total of 114 of 188 nurses working in the intensive care units of 3 private and 3 public hospitals in the province of Gaziantep in the Southeastern Anatolian Region of Turkey. Seventy four nurses were excluded from the study because they did not accept to participate in the research and did not meet the study criteria. Exclusion criteria included nurses working in the intensive care units for less than a year,^5^ pregnant nurses,^2^^,^^6^^,^^7^ and those with a metastatic disease^6^ and health problems that may cause low back pain. At all times, we made it clear that participation was voluntary.

***Definition of low back pain:*** Low back pain was defined as discomfort in the spinal area (between the lower costal margins and gluteal folds) experienced at least once a month ^3^, with or without radiation into the leg to below the knee.^2^

***Questionnaire:*** The study data were collected using a questionnaire prepared by the investigators with support from the literature.^1^^,^^3^^,^^8^ The questionairre validation and translation did not made. Only received the opinion of an expert for questionnaire. A pilot study was conducted with 18 nurses in a hospital not included in the research. It was tested there whether the survey questions were comprehensible and those that were not comprehensible were either excluded or revised. Filling out the questionnaire took about 15-20 minutes. The questionnaire included some descriptive characteristics of nurses, characteristics about low back pain, factors that may affect low back pain. Working hours were classified as day-time, watch system and shift. Watch system is defined as working at times outside official days and hours; shift is defined as working by turns outside normal day-time hours, during the day, in the evening or at night.^9^ In addition, low back pain was assessed by visual analogue scale (VAS; 0–10). In this scale, “0” indicated no pain and “10” indicated very severe pain.^10^

***Body mass index:*** Information on body weight and height were obtained from self-reports of nurses. Body Mass Index (BMI) was calculated as weight (kilograms) divided by square of height (meters); and values of 18.5 and lower were classified as “underweight”, 18.5 to <25 as “normal weight”, >25 as “overweight”, >30 as “obese”.^11^

***Ethical considerations:*** Consent was received from the nurses who were included in the study after they were provided with necessary explanation about the study’s objectives. Permission was received from the institutions where the research was conducted and approval was obtained from the Ethics Committee.

***Data analysis:*** Descriptive statistics were reported as frequencies, means and standard deviations, medians, and ranges. Chi-square was used to determine the relationship between characteristics of nurses, their low back pain status and factors that may influence having low back pain. Student t, ANOVA, Mann Whitney U and Kruskal–Wallis tests were used to determine the relationship between characteristics of nurses, factors that may influence their status of experiencing low back pain, and low back pain average scores. Correlation analysis was used to determine the relationship between the number of employment years, daily and weekly working hours, standing during working hours, and low back pain average scores. Statistically significant levels were set at p < 0.05.

## RESULTS

The relationship between specific characteristics of nurses and their status of experiencing low back pain is shown in Table-I. Although 84.2% of the nurses experienced low back pain and 66.7% of the nurses evaluated this pain as “moderately severe”, measurements performed by VAS revealed that the mean duration of low back pain was 1.5±0.8 years, and that 53.1% of the nurses experienced low back pain for 0-3 years and 39.6% had pain attacks once a week. It was determined that 37.5% of the nurses who experienced low back pain did not make any attempts to relieve the pain, 49.0% experienced an increase in low back pain, 71.9% did not see a doctor, and 79.2% did not receive any treatment for their low back pain (Table-II).

It was also determined that nurses who did not receive education on low back pain (86.5%), who stated that they sometimes complied with body mechanics during interventions to patients (56.3%), who remained standing for a long time (97.9%), who performed interventions that require bending forward (95.8%), who lifted patients (68.8%), who changed sheets while the patient was in the bed (65.6%), who repositioned patients (83.3%) and who did not use any aiding equipment during interventions (60.4%) experienced more low back pain (Table-III) and had higher mean pain scores (p>0.05) (Table-IV). Despite these observations, 85.4% of the nurses believed in the benefit of using aiding equipment during interventions. The nurses who were partially satisfied with their institution of employment (41.7%) were found to experience even more pain (p>0.05) (Table-III).

When the relationships between some of the characteristics of nurses and their mean pain scores were evaluated, it was found that the mean pain scores were higher in female nurses, those who were in the age group of 34 years and over, those who had master’s and doctorate degree, those with a chronic disease, those with a normal body mass index, those who worked in internal diseases and pediatric intensive care units, and those who worked in shifts (p>0.05) (Table-V).

In addition, correlation analysis revealed that low back pain score increased with the increase in years of employment, daily and weekly working hours, and duration of standing during working hours; but this result was not observed to be statistically significant (p>0.05).

## DISCUSSION

The studies evaluating low back pain in nurses showed that low back pain rates were higher ^2^^,^^7^^,^^12^^-^^16^ among nurses compared to other musculoskeletal system problems.^5^^,^^17^ In our study, it was observed that most of the nurses experienced low back pain, and this result was found to be similar to the findings of previously conducted studies. Therefore, it is important to define the risk factors that may lead to low back pain in nurses and to take the necessary protective measures. Nurses, who play an important role in protecting, maintaining and improving individuals’ health, should attach importance to applying protective and improving actions for their own health, so that they can provide nursing care quality, be productive, and administer patient care without interruption.^18^

**Table-I:** The relationship between specific characteristics of nurses and their status of experiencing low back pain

*Parameters*	*Those who experience low back pain n (%)*	*Those who do not experience low back pain n (%)*	*Significance*
*Gender*			
Male	7(7.3)	5(27.8)	p=0.022
Female	89(92.7)	13(72.2)	
*Age*			
18-25	27(28.1)	3(16.7)	p=0.095
26-33	56(58.3)	15(83.3)	
34 years and more	13(13.5)	-	
*Marital status*			
Married	52(54.2)	10(55.6)	p=0.561
Single	44(45.8)	8(44.4)	
*Children*			
Yes	57(59.4)	11(61.1)	p=0.554
No	39(40.6)	7(38.9)	
*Education*			
High school	21(21.9)	2(11.1)	p=0.762
Two-year university degree	16(16.7)	3(16.7)	
Bachelor’s degree	55(57.3)	12(66.7)	
Master’s/doctorate	4(4.2)	1(5.6)	
*Smoking *			
Yes	22(22.9)	4(22.2)	p=0.609
No	74(77.1)	14(77.8)	
*Wearing high-heeled shoes *			
Yes	39(40.6)	7(38.9)	p=0.554
No	57(59.4)	11(61.1)	
*Regular exercise *			
Yes	8(8.3)	2(11.1)	p=0.491
No	88(91.7)	16(88.9)	
*Body mass index*			
Poor	2(2.1)	1(5.6)	p=0.024
Normal	77(80.2)	9(50.0)	
Overweight	17(17.7)	8(44.4)	
*Evaluation of health condition*			
Very good	13(13.5)	3(16.7)	p=0.934
Good	54(56.3)	10(55.6)	
Moderate	29(30.2)	5(27.8)	
*Working status *			
Head nurse	10(10.4)	1(5.6)	p=0.453
Nurse	86(89.6)	17(94.4)	
*Clinic of employment*			
Surgery intensive care	38(39.6)	10(55.6)	p=0.269
Internal medicine, intensive care	23(24.0)	1(5.6)	
Coronary intensive care	14(14.6)	3(16.7)	
Pediatric intensive care	17(17.7)	2(11.1)	
Reanimation	4(4.2)	2(11.1)	
*Working status *			
Day-time	35(36.5)	6(33.3)	p=0.137
Shift	34(35.4)	3(16.7)	
Watch	27(28.1)	9(50.0)	
*Night work in the last one year*			p=0.458
Yes	70(72.9)	14(77.8)	
No	26(27.1)	4(22.2)	
*Duration of employment (year) Transportation to work*	6.7±4.4	5.8±2.9	p=0.563
Walking-bicycle	27(28.1)	5(27.8)	p=0.996
Public transportation	47(49.0)	9(50.0)	
Private car	22(22.9)	4(22.2)	
Total	96(100.0)	18(100.0)	

**Table-II:** Distribution of low back pain-related characteristics of nurses who experience low back pain

*Parameters*	*n(%) *
*Low back pain severity*	
Mild Moderate Severe	25(26.0) 64(66.7) 7(7.3)
*Low back pain duration*	
0-3 years 4-6 years 7-10 years 11 years and more	51(53.1) 27(28.1) 13(13.5) 5(5.2)
*Low back pain frequency*	
All the time Once a week Once a month More than once a month After shifts	25(26.0) 38(39.6) 18(18.8) 14(14.6) 1(1.0)
*Interventions performed to overcome low back pain *	
Nothing Exercise Massage Resting Medication Other	36(37.5) 14(14.6) 3(3.1) 26(27.1) 8(8.3) 9(9.4)
*Experiencing an increase in low back pain*	
Yes No	47(49.0) 49(51.0)
*Seeing a doctor for complaints of low back pain *	
Yes No	27(28.1) 69(71.9)
*Receiving treatment for low back pain*	
Yes No	20(20.8) 76(79.2)
Total	96(100.0)

**Table-III:** The relationship between the nurses’ status of experiencing low back pain and the influencing factors

*Parameters*	*Those who experience low back pain n(%)*	*Those who do not experience low back pain n(%)*	*Significance*
*Receiving education on low back pain*			
Yes No	13(13.5) 83(86.5)	5(27.8) 13(72.2)	p=0.124
*Complying with body mechanics during interventions *			
Yes No Sometimes	10(10.4) 32(33.3) 54(56.3)	4(22.2) 6(33.3) 8(44.4)	p=0.348
*Standing for a long time*			
Yes No	94(97.9) 2(2.1)	18(100.0) -	p=0.708
*Doing works that require bending forward*			
Yes No	92(95.8) 4(4.2)	18(100.0) -	p=0.498
*Lifting patients* Yes No	66(68.8) 30(31.2)	14(77.8) 4(22.2)	p=0.321
*Bathing patients*			
Yes No	34(35.4) 62(64.6)	5(27.8) 13(72.2)	p=0.368
*Changing sheets while the patient is in the bed*			
Yes No	63(65.6) 33(34.4)	12(66.7) 6(33.3)	p=0.581
*Changing patients’ clothes*			
Yes No	36(37.5) 60(62.5)	7(38.9) 11(61.1)	p=0.555
*Repositioning patients*			
Yes No	80(83.3) 16(16.7)	13(72.2) 5(27.8)	p=0.211
*Pushing-pulling heavy objects*			
Yes No	48(50.0) 48(50.0)	15(83.3) 3(16.7)	p=0.008
*Using aiding equipment during interventions *			
Yes No	38(39.6) 58(60.4)	9(50.0) 9(50.0)	p=0.285
*Benefit of using aiding equipment during interventions*			
Yes No	82(85.4) 14(14.6)	17(94.4) 1(5.6)	p=0.269
*Satisfaction with the institution of Employment*			
Yes No Partially	38(39.6) 18(18.8) 40(41.7)	7(38.9) 7(38.9) 4(22.3)	p=0.227
Total	96(100.0)	18(100.0)	

**Table-IV:** The relationship between specific parameters that may affect low back pain in nurses and their mean low back pain scores

*Parameters*	*Pain* *Mean±SD*	*Significance*
*Complying with body mechanics during interventions*		
Yes No Sometimes	1.3±1.0 1.4±0.7 1.5±0.8	p=0.757
*Receiving education on low back pain*		
Yes No	1.2±0.8 1.5±0.8	p=0.228
*Duration of low back pain*		
0-3 years 4-6 years 7-10 years 10 years and over ↑	1.3±0.9 1.7±0.5 1.8±0.5 1.8±0.4	p=0.164
*Lifting patients*		
Yes No	1.5±0.8 1.5±0.7	p=0.074
*Standing for a long time*		
Yes No	1.5±0.8 1.1±0.8	p=0.074
*Bathing patients*		
Yes No	1.5±0.8 1.5±0.8	p=0.729
*Changing sheets while the patient is in the bed*		
Yes No	1.5±0.8 1.4±0.7	p=0.552
*Changing patients’ clothes*		
Yes No	1.5±0.9 1.5±0.7	p=0.932
*Pushing-pulling heavy objects*		
Yes No	1.4±0.9 1.5±0.6	p=0.478
*Repositioning patients *		
Yes No	1.5±0.8 1.3±0.8	p=0.242
*Using aiding equipment during interventions *		
Yes No	1.4±0.8 1.5±0.8	p=0.396
Total	1.5±0.8	

**Table-V:** The relationship between specific characteristics of nurses and their mean low back pain scores

*Parameters*	*Pain* *Mean±SD*	*Significance *
*Gender*		
Male Female	1.0**±**0.9 1.5±0.8	p=0.104
*Age*		
18-25 26-33 34 and more	1.5±0.6 1.4±0.9 2.0±0.4	p=0.083
*Education*		
High school Two-year university degree Bachelor’s degree Master’s/doctorate	1.7±0.6 1.5±0.8 1.4±0.8 1.8±1.3	p=0.256
*Wearing high-heeled shoes *		
Yes No	1.4±0.8 1.5±0.8	p=0.464
*Regular exercise*		
Yes No	1.5±0.9 1.5±0.8	p=0.951
*Chronic disease*		
Yes No	2.0±0.0 1.4±0.8	p=0.098
*Body mass index*		
Poor Normal Overweight	1.0±1.0 1.5±0.7 1.3±1.0	p=0.359
*Health condition*		
Very good Good Moderate	1.4±0.8 1.5±0.8 1.5±0.8	p=0.741
*Clinic of employment*		
Surgery intensive care Internal diseases intensive care Coronary intensive care Pediatric intensive care Reanimation	1.5±0.9 1.6±0.6 1.5±0.7 1.6±0.8 1.0±0.8	p=0.980
*Working condition*		
Day-time Shift Watch	1.5±0.8 1.7±0.7 1.3±0.8	p=0.226
Total	1.5±0.8	

In our study, most of the nurses worked in standing position for long durations, performed interventions that required bending forward, lifted and repositioned patients, and these nurses had higher average low back pain scores. Moreover, it was observed that the nurses who did not use any aiding equipment during interventions yet believed in the benefit of using it constituted the majority. It is a striking result that although nurses frequently performed interventions that may create risk factors for low back pain, such as standing for long durations, performing interventions that require bending forward and lifting and repositioning patients, and although they knew the proper application in using aiding equipments; they did not reflect this knowledge in their interventions. This result may be explained by the lack of sufficient education given to nurses about the risks that may cause low back pain and the lack of sufficient time during interventions for using aiding equipment.

The studies conducted on low back pain have demonstrated that there is a relationship between smoking and low back pain,^19^ and that smoking impairs nutrition in the disk, making it more vulnerable against outside influences and disturbing blood flow.^18^^,^^20^ However, as in our study, there are also study that found no relationship between smoking and low back pain.^6^ Our findings may be explained by the low number of non-smoker nurses included in the research. 

In previously conducted study, a relationship was found between gender and low back pain, and women were shown to experience more low back pain.^8^ This result may be associated with the anatomic, physiologic and structural difference between the sexes, and the low number of male nurses included in our study. Our study also revealed a statistically significant relationship between low back pain and education status among other socio-demographic variables, and showed that nurses with master’s and doctorate degrees had higher low back pain average scores compared to others. A similar result was obtained in another study. in our country, and this result was associated with the fact that those with higher education level spared more time for patient care and gave more prominence to their professional roles.^19^ On the other hand, in another study conducted in our country, no significant relationship was found between education level and low back pain.^18^

In our study, work conditions such as working hours and institution of employment were determined to be effective on low back pain. It was reported that low back pain increased in parallel with the increase in working hours and this result was associated with sparing less time for resting.^15^ In another study it was assessed that relationship between the clinic of employment and low back pain. It was observed that the orthopaedic and ICU departments have heavy workloads that are likely to cause low back pain. Therefore it was suggested, the clinics in which nurses work and the risks posed by these clinics should be evaluated.^1^ In our study, it was observed that especially the nurses who worked in internal diseases and pediatric intensive care units had higher low back pain average scores. Similarly, a higher prevalence of low back pain was also reported among nurses working in these clinics by other researchers.^3^ Higher low back pain average scores observed in nurses working in internal diseases and pediatric intensive care units may be associated with the fact that interventions that are more likely to cause low back pain are applied more in patients hospitalized in these units since these patients need different nursing cares, and that these clinics provide service under different conditions.

In addition, it was also found that working conditions and satisfaction with the place of employment affected low back pain; nurses who worked in shifts had higher low back pain average scores; and nurses who were partially satisfied with their place of employment experienced more low back pain. Working with fewer personnel during shifts, having to perform patient transfers on one’s own without help, lack of sleep, and decrease in the quality of sleep may be associated with low back pain. Moreover, it is thought that the employees feel better and experience less anxiety as their satisfaction with the institution of employment increase, and that these factors have a positive effect on low back health. There are studies that determined a relationship between low back pain and working conditions and satisfaction with the place of employment;^21^ whereas, no relationship was found in some studies between these factors and low back pain.^18^ Furthermore, in our study, it was found that nurses who evaluated their health condition as “very good” experienced less low back pain problems and had lower low back pain average scores. This result may indicate that nurses who define their health status as “very good” feel much better, as observed in the satisfaction with the institution of employment, and this situation increases their performance and therefore decreases their low back pain related problems. This observation is also supported by the study conducted by Alexopoulos et al., which revealed that those with worse self-perceived health condition experienced more low back pain.^16^

In line with these results; it may be suggested that regular education programs should be initiated in intensive care units in order to control risk factors that may cause low back pain; nurses should be provided with guidance on using aiding equipment that would reduce physical load; and necessary protocols should be established to control compliance to these rules by close monitoring. In addition, it is considered highly important that necessary attention is paid to complying with body mechanics during all kinds of nursing interventions in patient care, and the differences between clinics in terms of the risk factors for low back pain are taken into consideration.

## Authors Contribution:

OO, NO, MG: Study design. MG: Data collection.

OO: Data analysis. OO, NO, NCA: Manuscript preparation.

## Notes

***Limitations of the study*** *: *This study has several limitations. First, our study was cross-sectional in design; the sample size was small; and this study was conducted only in the institutions in Gaziantep province located in the Southeastern Anatolia Region of Turkey. Thus, the results and conclusions should not be generalized for nurses in all intensive care units in Turkey. Secondly, study data were collected by using questionnaires, without observation.

## Notes

***Source of Funding:*** None.
